# Reverse chronology quota record screening for realist synthesis: Fostering causally rich extrapolations with a diverse and contemporaneous sample of literature

**DOI:** 10.1017/rsm.2025.10068

**Published:** 2026-01-26

**Authors:** Justin Jagosh, Simon Briscoe, Sarah Greenley, Adrian Edwards, Frederikus Klok, Miriam Johnson, Simon Noble, Mark Pearson

**Affiliations:** 1 Hull York Medical School, University of Hull, UK; 2 College of Medicine and Health, Institute of Health Research, University of Exeter, UK; 3 Division of Population Medicine, Cardiff University, UK; 4 Thrombosis and Hemostasis, Leiden University Medical Center, Netherlands

**Keywords:** article selection, identification, realist review, realist synthesis, screening

## Abstract

Identifying studies for inclusion in realist syntheses using title and abstract screening is challenging given the need to unearth explanatory insights to build context–mechanism–outcome configurations. Such insights may only be uncovered through full-text paper reading. To address this issue, a novel approach for identifying studies has been developed called *Reverse Chronology Quota Record Screening* (RCQRS). Results of database searches are screened in reverse chronology, and in stages, to fill quotas matching the requirements of the review. RCQRS may be beneficial in any of the following circumstances: (a) the timeframe to complete the synthesis is short; (b) the scope of inquiry is not fully defined, (c) the availability of suitable literature is beyond the screening capacity of the reviewers; *or* (d) the availability of literature is sparse and reviewers seek to extrapolate insights from other areas. In contrast to RCQRS, exhaustive title and abstract screening (i.e., screening entire database results) may hamper study selection due to being overwhelming in volume and time-intensive, resulting in a causally thin cohort of papers for analysis. RCQRS used in stages, and in conjunction with other search strategies (e.g., hand searching, backward citation tracking, and expert solicitation) can support creative, robust analytical insights with causally rich extrapolations. Using the Horizon-EU funded SERENITY study on deprescribing in palliative care as a case example, the benefits and limitations of RCQRS are explored. Finally, a checklist template is offered for teams who wish to reflect on, and transparently report, the use of RCQRS in their realist synthesis.

## Highlights

### What is already known?

Exhaustive Record Screening, in which all records from a database search are screened before analysis, is the standard way in which study selection is accomplished in most review methodologies including realist syntheses.

### What is new?

Reverse Chronology Quota Records Screening is an innovative, efficient way to accomplish study selection in a realist synthesis, potentially more in line with the philosophy of realist methodology and the iterative, complexity-focussed needs of the realist analysis.

### Potential impact for RSM readers

Readers may consider RCQRS as an approach to innovate the design of their realist syntheses. RCQRS may also be explored for other types of reviews.

## 1 Introduction

Timely identification of studies for inclusion in research syntheses is a daunting task, particularly in reviews that investigate complex research areas with explanatory questions about the causal functioning of intervention efforts. Realist synthesis is such an approach that addresses the questions ‘what works, for whom, under which circumstances, and how’.^1^ This methodology can incorporate a variety of search strategies for identifying studies^2^ including the use of bibliographic databases, and supplementary search methods such as hand searching and soliciting papers through field experts. A variety of data sources can also be considered,^3^ including primary research, other reviews, and non-empirical literature, such as reports, commentaries, and social media. Outputs of a realist synthesis are framed as retroductive insights (i.e., context-sensitive ‘how’ and ‘why’ statements)^4^ on the architectural features of interventions, including their causal mechanisms.^5^

Realist logic of inquiry challenges the binary, often positivist, framing of ‘effectiveness’ and posits that interventions will typically have partial success and failure across differences in stakeholder groups, contexts, and timepoints. Exploring these non-uniform results and their mechanisms is key to improving the customisation of intervention efforts.^6^^,^ ^7^ The context-sensitive approach in realist methodology informs the explanatory understanding of programme functioning.^8^ Given that not all evidence from a cohort of papers will contain explanatory insight, the realist synthesis can also be used to build new theoretical insights inspired by evidence,^9^ requiring creative (abductive) thinking, co-production with diverse stakeholders, and a transparent, rigorous process in analysis.^4^ This principle is underscored by a quote from Pawson et al.,^10^ which emphasises the importance of using realist synthesis as a way of improving thinking around the design of programmes and services:

*With its insistence that context is critical and that agents interact with and adapt to policies and interventions, realist synthesis is sensitive to diversity and change in programme delivery and development. Its fundamental purpose is to improve the thinking that goes into service building. And in doing so, it provides a principled steer away from issuing misleading ‘pass/fail’ verdicts of entire families of interventions and away from failed ‘one-size-fits-all’ ways of responding to problems* ^10^

*(p. iii)*

This line of argument is furthered in Pawson and Bellamy,^1^ emphasising the need for literature review to move past summarising evidence and to support ‘fresh thinking’ in policy and programme development:

*Some knowledge gain, some novel compound, some added value is produced in the process of synthesis. Such a notion is also vital to evidence-based policy. There is a need for systematic review to go beyond reportage and summary of an existing state of affairs. The point, after all, is to support fresh thinking to revise policy and launch it in new circumstances’* ^1^

(p. 73).

Supporting fresh thinking is important, as it is often the case that realist syntheses are completed in areas with pre-existing and prolific trails of research and where general knowledge on the topic is well established. In advancing the purpose of realist synthesis, and optimising the outputs, we introduce a novel and efficient screening technique called Reverse Chronology Quota Record Screening (RCQRS). The technique has been conceived by first author (JJ) and used in two published projects including the one described in this article.^11^^,^ ^12^ All co-authors have been involved in one of these realist synthesis projects using RCQRS. Co-authors SB, MP, and SG have been directly involved in the development of the approach.

## 2 What is RCQRS?

RCQRS is an approach to screening the results of bibliographic database searches by organising the retrieved database records in reverse chronology (i.e., starting the screening with the most recent publication record). Screening is conducted using an inclusion/exclusion tool that is responsive to the review questions and initial programme theories. Screeners read and deliberate on the inclusion of records until a pre-established, relatively small quota category is (or a set of quota categories are) filled. Once quotas are filled, the full-text papers are retrieved and analysed using realist logic. After the completion of an initial rough analysis, reviewers can consult stakeholders and reflect on the quality and representation of data against programme theories, and use the new learnings gained to: (a) return to the database record library to continue screening in reverse chronology from the last record screened, (b) conduct sub-searches of the existing database using new quota categories based on the initial analysis, or (c) conduct new database searches altogether.

RCQRS works best by screening papers in small batches, analysing the literature in stages, and including papers from other approaches such as hand searching and expert solicitation. This technique is comparable to traditional searching and screening approaches that are designed to manage large yields in the database record library and truncate screening, including date-limits and stopping rules.^13^^,^ ^14^ Date-limits are filters that retrieve records within a certain publication timeframe. Stopping rules are often based on establishing a pre-specified number of results once reviewers no longer see any new relevant content, typically in cases where the results are ordered in terms of relevance (e.g., search engines, such as Google Search or Google Scholar). RCQRS is different from these approaches in that it prioritises contemporaneous publications and supports the inclusion of roughly relevant papers, as well as ‘highly matched’ papers, for extrapolations using realist logic.^10^ RCQRS does not require the use of date-limits, and there may be some benefit to establishing searching parameters without date-limits. Although RCQRS without date-limits will create a larger database record library than will be used for systematic screening given the reverse chronology quota approach, it may still be advantageous to retain a large database library from the search. This is because reviewers may wish to conduct sub-searches within the full database record library in later stages of the review, as new priority areas emerge through analysis and consultation with stakeholders.

### 2.1 RCQRS versus exhaustive record screening for realist synthesis

RCQRS can be contrasted with exhaustive record screening (ERS), which is used in most realist syntheses. Using ERS, reviewers screen every record retrieved by the database search after duplicates are removed and include those that appear to best address the research question before full-text paper reading and analysis. This involves an inclusion/exclusion tool with screening questions (see, e.g.^15^) or a traffic-light system for screening (i.e., green ‘include’, yellow ‘maybe’, and red ‘exclude’).^16^ Even with strictly applied screening tools, ERS may still yield more papers suitable for full-text reading than is possible to use (e.g., >500). This is particularly relevant when new and important aspects of context emerge through the screening process. When there are too many papers to read at the full-text reading stage, realist reviewers often apply new narrowing criteria, including limitations to intervention type, geographical area, or social context, to further reduce the number to a manageable size. Eliminating diversity from the retained sample can detract from building creative extrapolations and a holistic view of programmatic efforts. Furthermore, contrasting the content of diverse literature can support the articulation of mechanisms, which are often deep and hidden from view.^4^^,^ ^17^ Using a theoretical example, Table 1 presents a summary of this problem:

**Table 1:** How large-volume record screening is often managed in a realist synthesis Table 1 Long description.

Example framing of a realist synthesis question: *‘What works, for whom, and in what circumstances for drug addiction recovery?’*	
Narrowing to…	Explanation
…a subset of interventions	Choosing to retain only one type of interventions even if there are many types of interventions addressing the problem in question. For example, choosing peer-led interventions for drug addiction recovery and excluding provider-led interventions
…a subset of contexts	Choosing to retain only those programmes targeting a certain setting or demographic characteristic when the need to understand mechanisms can benefit from a broader range of contexts. For example, choosing drug addiction recovery interventions for persons experiencing homelessness and excluding all other drug addiction circumstances
… a subset of study design and literature source	Choosing to retain only one type of study design such as systematic review or randomised control trial (RCT) while excluding or de-prioritising all other data sources. For example, only retaining RCTs on drug addiction recovery interventions for persons experiencing homelessness and excluding case study design, qualitative research and commentaries

## 3 How RCQRS is beneficial for realist synthesis

Realist synthesis harnesses causal insights from the literature to build context–mechanism–outcome (CMO) configurations.^18^ Such causal insights are not often described in the paper’s title and abstract and therefore, screeners have limited means to deliberate on papers at the screening stage. Given this challenge, RCQRS allows for a more rapid screening stage and an earlier start to data analysis, which allows more time for full-text explorations of papers.

It is often the case that realist syntheses are completed in areas in which there has been a long and prolific trail of research and where general knowledge on the topic is well-established. Given this reality, the lengthy screening timeframes needed for ERS lead to shortened timeframes for analysis, which risk repeating the similar conclusions of previous research while imposing realist concepts (i.e., CMO) on already well-established assumptions. This risks significantly limiting the explanatory potential of realist synthesis and inhibiting scientific accumulation of knowledge. The issue of creating superficial, banal conclusions from realist research has previously been identified in the realist community of practice and requires concerted effort to address.^22^^–^ ^24^ The complexity of problems being addressed through realist thinking requires synthesis design considerations that reduce summarising and repeating conclusions of past research and rather increase creative (i.e., abductive)^25^ explorations to generate new understandings in areas of inquiry. This consideration is ever more important in any of the following circumstances: (a) the timeframe to complete the realist synthesis is often short and therefore not amenable to an exhaustive screening of the literature, (b) the scope of inquiry is not fully defined and thus a more exploratory approach is needed, (c) the availability of suitable literature in relation to the review questions is vast and beyond the screening capacity of the reviewers which would overwhelm the screening process and cause decision fatigue in the screeners, or (d) the availability of suitable literature is sparse and reviewers are seeking to extrapolate insights from other areas, which can also rapidly proliferate the options for paper inclusion and overwhelm the screening process. Table 2 provides a definition of terms for this discussion. Potential advantages and disadvantages of the approach are included in Table 3.

**Table 2:** Definition of terms Table 2 Long description.

**Database Record:** A collection of information about a published paper including title, abstract, authorship, date, journal of publication, and location
**Database Record Library:** The complete set of records retrieved from a database search after duplicates have been removed.
**Database Searching:** the process of identifying key search terms and entering these into bibliographic databases which index records of published empirical and non-empirical papers
**Exhaustive Record Screening:** The process of reading and adjudicating on every record produced through the database search after duplicates are removed. ERS means all screening is complete before included records are used to retrieve and read the corresponding full-text paper
**Expert Solicitation:** Contacting experts including researchers, practitioners and service-users to recommend literature that they believe would be beneficial to be included in the review
**Hand Searching:** The process of selecting key academic journals, scanning the table of contents to identify potentially relevant papers for inclusion in the review
**Reverse Chronology Quota Record Screening:** The process of screening database records in reverse chronology from the most recent publication date until quotas of suitable papers have been filled
**Screening:** the process of reading database records and deliberating on the suitability of the records for including in the synthesis
**Study Selection:** The full set of strategies including screening and critical appraisal that allow reviewers to deliberate on and retain a group of papers for realist analysis^19^

**Table 3:** Advantages and limitations of RCQRS Table 3 Long description.

Advantages	Explanation
1. Improved pacing of review stages and estimations of time required to completion	Setting quotas for screening can improve pacing of review activities across the timeline. This can inspire confidence of the reviewers that the review is going to be a manageable endeavour
2. Increased time for data immersion and analysis	RCQRS shortens the timeframe for selecting papers which provides more time for data immersion and analysis. Whereas an exhaustive approach to screening titles and abstracts in a systematic review may take upwards of 1000 hours,^20^ RCQRS with broader inclusion criteria may require roughly 25–50 hours
3. Reduced decision fatigue at screening stage	Screening large volumes of records for inclusion/exclusion (e.g., >15,000) can feel unsettling for screeners, given that important causal insights are often buried in papers and not knowable from title and abstract reading. RCQRS reduces decision over-labouring for record screening and allows reviewers to move quickly to read full-text papers for further adjudication
4. Improved representation of diversity in context and programme architecture through multiple quota categories	Realist reviewers may be expected to provide analysis on how multiple interventions work for a variety of setting and demographic contexts. Quota categories can ensure that an adequate number of papers are retained for the different areas of importance, ensuring representation from under-represented or under-researched areas of importance
5. Retention of papers that are ‘roughly relevant’ to the review topic supports new discovery and creative insights	Quicker timeframes for screening allow for the inclusion of roughly relevant papers for an exploratory approach to causal insight extrapolation. Such papers with tangential relevance may have surprising richness that supports abductive theorising.^21^ Stakeholders and co-investigators involved in data analysis may find it conceptually refreshing to work beyond the overly familiar, tried-and-(apparently) true conclusions of previous well-known research on the topic and experience intellectual growth from diversity in study selection
6. Studying Programmes in Most Recent Contexts, Circumstances and Settings	RCQRS captures the most recently published papers reflecting most recent contexts and programme innovations and theories, increasing the pragmatic utility of the findings for current challenges in the intervention area under scrutiny
Limitations	Explanation
1. Potential to miss important papers after quotas are filled from the database record library	Using RCQRS means that only a portion of the database record library will be screened and the rest will be left unexamined. It is likely that useful, relevant papers will not be included in the dataset for this reason. Therefore, combining RCQRS with other strategies will help to mitigate the potential loss from not screening the entire database record library
2. Set of papers retained using RCQRS is too diverse to analyse for specific review questions	RCQRS will likely capture roughly relevant papers which may be tangentially related to the review questions. Such an approach may not be suitable for the novice realist reviewer, or for some reviews in which the focus is very specific and conducting an exhaustive search of the literature may be advantageous. Some searches result in very manageable screening tasks due to low numbers of records retrieved in the database record library. In this case, an exhaustive approach to screening may not take more time than RCQRS and may be advantageous for that reason
3. Combining RCQRS with expert solicitation may introduce papers which are not relevant, or are too specific	Using RCQRS with expert solicitation may mean that experts recommend papers that they prefer, even if they do not align with the goals of the review or do not provide rich insights into the explanatory mechanisms of programmatic efforts. In this case, these papers may only serve as indirectly supportive of the review and incorporating them may cause inefficiency in the review process

## 4 Case example: The SERENITY realist synthesis

The *SERENITY* study realist synthesis examined mechanisms of deprescribing anti-thrombotic therapy (ATT) for patients living with cancer in their last phase of life.^11^^,^ ^26^ The study was part of the larger research project called ‘Towards Cancer Patient Empowerment for Optimal Use of ATT at the End of Life’ (https://serenity-research.eu/). People living with cancer undergoing treatment are at increased risk of thrombotic complications and are usually provided with ATT to mitigate this risk. However, physiological changes arising from late-stage cancers increase the possibility that ATT will cause clinically relevant bleeding and impact quality of life. Additionally, ATT may cause drug-drug interactions leading to adverse reactions, interfere with the efficacy of other medications, and cause overall medication burden. While improved understanding of patients’ preferences supports optimal ATT prescribing, clinicians seldom discuss the possibility of deprescribing ATT with end-of-life patients or engage in risk–benefit decision-making. Patients often needlessly remain on the medication until death and with adverse outcomes and experiences.

To better understand the reasons underpinning prescribing continuance in this context, this realist synthesis was undertaken to broadly explore literature on shared decision-making (SDM) and deprescribing in end-of-life care and to extrapolate findings to build a conceptual platform for optimising the use of ATT for persons living with cancer in the last phase of life. Given a paucity of research papers with a specific focus on SDM and deprescribing for ATT in patients with cancer in their last phase of life, the realist synthesis examined a diverse range of papers broadly related to the issues of deprescribing and SDM in palliative care. The RCQRS approach allowed for rapid retention of papers for full-text reading and causal extrapolations to the specific question of ATT deprescribing. In doing so, the conclusions of the realist synthesis yielded new insights about how the meaning that medications have to patients will impact on clinicians’ volition to deprescribe, especially in the last phase of life and that different classes of medications require a customised approaches to SDM with patients. Although no papers were found which were specific to SDM for ATT deprescribing, the review produced recommendations specific to the area of deficit that was being investigated.

Database searching across 10 databases resulted in the retrieval of a database record library involving 17,036 citations. RCQRS was applied at an initial stage to capture 10 papers on SDM in palliative care, and an additional 10 papers on deprescribing in palliative care. A total of 230 records were screened to achieve this initial retained set and an initial rough analysis of these papers was completed and shared with team members. Two stakeholder consultation meetings were held at this stage with practitioners and members of the public to discuss insights gained from this preliminary analysis. From these discussions, additional areas of inquiry were identified including organisational factors affecting deprescribing efforts and moral injury of staff witnessing severe bleeding events in patients in their last phase of life due to ATT prescription. An additional 257 records were identified from the database record library and screened with a final retention of 56 papers from database searching and an additional 35 papers (irrespective of date) solicited from consortium experts. Further reflection on the use of RCQRS in the SERENITY project is found in the sections below. Full details on the study, including protocol, realist analysis, and results, are reported elsewhere.^11^^,^ ^26^

## 5 Five steps in the process of using RCQRS

**Figure 1:**
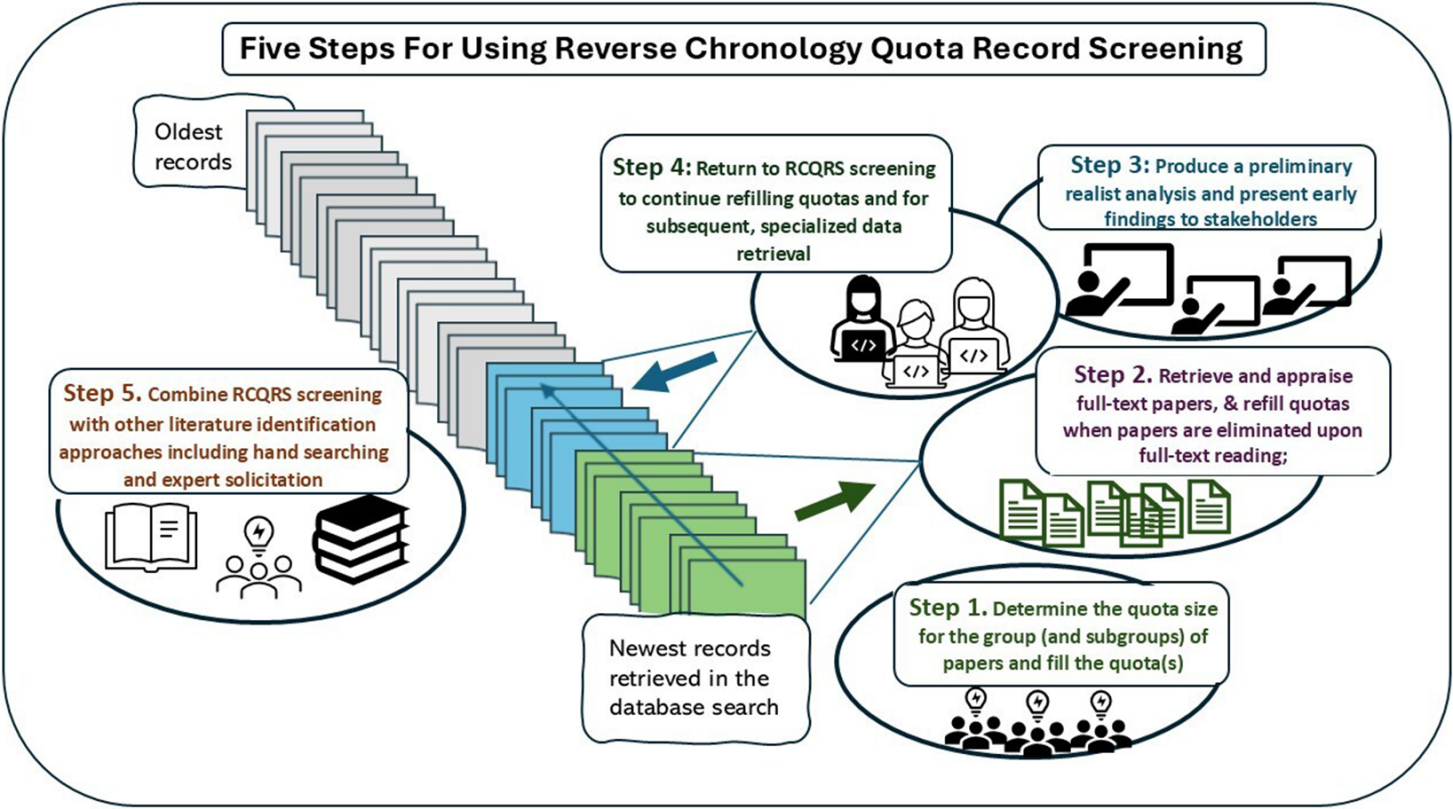
Five Steps for Using Reverse Chronology Quota Record Screening. Figure 1 Long description.

The process for using RCQRS is explained across five steps, using the SERENITY review experience to illustrate the points. Figure 1 provides a snapshot view of the steps. Table 4 and the section below outline the steps and the process in greater detail. A checklist template is offered in Appendix 1 (see Supplementary Materials) for teams who wish to reflect on and transparently report their use of RCQRS in their realist synthesis. This template can be used at the protocol development stage and revisited during stages of the review when iterative modifications or additions to the screening strategy are undertaken.

**Table 4:** Five steps for using RCQRS within a realist synthesis Table 4 Long description.

Step 1: Determine the quota size(s) for the numbers and screen records to the quota limits
Step 2: Retrieve and appraise full-text papers, and refill quotas when papers are eliminated upon full-text reading
Step 3: Produce a preliminary analysis and present early CMO configurations to stakeholders
Step 4: Return to RCQRS after preliminary analysis to continue refilling quotas and for specialised data retrieval
Step 5: Combine RCQRS with other literature identification sources including hand searching and expert solicitation

## 6 Step One: Determine the quota number limits for the group and subgroups of papers and screen records to the quota limits

The first step in using RCQRS is to establish a quota number and limit for the synthesis overall, and sizes for the sub-quotas (i.e., categories) (if any), which may map to different programme types and contexts. Depending on time and resources, a decision can be made to conduct screening with a single screener, or for two screeners to undertake partial or 100% coverage of content to be screened (and for which inter-rater reliability can be calculated).^27^

Three considerations can be used to determine quota size. The first is the degree of clarity reviewers have on the scope for the review and the architecture of programme(s) under investigation. A lack of clarity in these areas may mean that many papers deemed relevant will overwhelm the screening process. Thus, choosing a small quota size as an initial step (e.g., n = 15) allows the reviewers to begin reading and analysing full-text papers early in the review to explore the content for programme descriptions, causal insights, and CMO configurations. This early appraisal will help to determine whether the content of the papers retained is indeed yielding the kinds of insights needed to address the review questions.

The second consideration for determining the size of the quota is the amount of time and resource allocated for the review. For example, a 6-month allocation for the synthesis may be suitable for a maximum of 60 papers in the review, provided all papers are rich and relevant to the review questions and initial programme theories. Alternatively, if the review has a longer duration (e.g., an 18-month duration), the team may consider a larger quota (e.g., 90–120 papers), which can increase the scope, complexity, comprehensiveness, and ability of the review to inform theoretical outputs. Although there are no firm guidelines on the number of papers to be included, these estimates may serve as a rough guide.

A third consideration is to have multiple quota categories for various contexts and variations in programme architecture. This can ensure representation across important aspects under review. For example, a review examining mechanisms of support for dementia care workers may include a quota category for homecare support, nursing homes, and via hospice services, with equal (or equitable) numbers to fill in each. Quota categories can also be established according to data source. For example, a separate quota for systematic reviews and non-empirical papers, such as editorials, can ensure that the ratio of primary empirical studies to additional sources is by design and not by happenstance. The inclusion of systematic reviews can also be used to retrieve older seminal papers through purposeful sampling, which would otherwise be missed. Quota categories can thus ensure the richness of the retained dataset and ensure representation of both content and research type. See Supplementary Appendix 1 for further guidance.

A suitable screening application such as RAYYAN, EPPI reviewer, or Endnote database files can be used, so long as whichever application is used has the option to order the records in reverse chronology.

### 6.1 SERENITY reflection

The SERENITY realist review had a 12-month timeframe. Preliminary scoping of the literature indicated a dearth of studies with the focus on SDM for deprescribing ATTs for persons living with cancer in their last phase of life. Therefore, a decision was made to broaden the scope to examine papers more generally on SDM in palliative care, and papers on deprescribing in palliative care, and to extrapolate from the findings of those papers to the specific research questions. The main search involved 10 databases and yielded 17,036 citations. An initial quota of 10 papers each in both SDM and deprescribing was reached in 2 days. 230 records were screened to reach those quota limits for both categories. All screening was conducted in RAYYAN due to the facility of ordering the citations in reverse chronology in that application. At the time of conducting this review, Covidence did not offer a feature to order records in reverse chronology.

## 7 Step Two: Retrieve and appraise full-text papers, and refill quotas when papers are eliminated upon full-text reading

Once the quotas for screening have been reached, full-text papers are retrieved, read, and appraised for relevance, richness, and rigour.^1^^,^ ^28^ It is usual for several papers in the quota to be excluded on account of the appraisal judgement after full-text reading. Reviewers can then return to the database record library at the last record screened and continue screening in reverse chronology until replacement papers are found to refill the quotas.

### 7.1 SERENITY reflection

The first 20 papers were read and analysed through an appraisal journal that was co-produced with the co-investigative team. All papers in these batches were deemed relevant, and no quota refilling was required. The journal entries were progressed to a rough preliminary analysis and included reflections on the causal significance of the paper content for the SERENITY review questions, along with exploratory realist ‘if…then’ statements and CMO configurations. The rough analysis was then vetted by the co-investigative team in advance of consultation meetings with stakeholders.

## 8 Step Three: Produce a preliminary analysis and present early CMO configurations to stakeholders

From the papers that have been appraised and deemed acceptable for inclusion in the analysis, reviewers then proceed to analyse these papers, coding for causal insights and CMO configurations. Initial programme theories may be scrutinised against their contents and the analysis can then be presented to the wider co-investigative team, practitioner consortia, and patient and public advisory groups. These stakeholders will likely be able to provide input in terms of confirmation, refutation of the early findings, and recommendations for new or refined directions in the review.

### 8.1 SERENITY reflection

The findings from this phase were presented at two consultation meetings in month 5 of the review, one with a practitioner consortium and another with patients and members of the public. Select passages of content from papers were presented to stakeholders along with realist programme theories. Stakeholders provided confirmation on the direction of the research progress and identified new areas for investigation. For example, one area of importance identified at the first practitioner consultation meeting was the need to better understand organisational factors that support or impede clinicians from engaging in SDM and deprescribing with persons living with cancer in their last phase of life. Another issue practitioners raised was the fact that different specialists may disagree on the need for or timing of deprescribing. These and other insights were taken back to the review team for a second phase of searching.

## 9 Step Four: Return to RCQRS after preliminary analysis for subsequent, specialised data retrieval

Once a preliminary analysis of papers and stakeholder vetting has been conducted, it is likely that the learnings achieved during this phase will prompt new questions for the review, which can be used for subsequent searching. These learnings may also arise by presenting preliminary findings to stakeholders who can then identify gaps in the current set of papers. Resuming bibliographic database searching and title and abstract screening can involve conducting entirely new database searches with new keywords and applying RCQRS to these new searches, or using the original search results and re-searching within the original database record library. This decision will depend on how extensive the original database search is and whether new areas for exploration are represented within the original search strategy. Such iterative searching is only made possible by the shortened timeframes of the initial searching arising from RCQRS. However, only a few cycles of iteration will be possible within the average timeframe of conducting a funded review, such as 12 or 18 months. More iterative searching will be possible with increased time and resource allocation for the review.

### 9.1 SERENITY reflection

Based on feedback by SERENITY project stakeholders, new keywords were developed to search the existing database record library (n=17036). Additional quota categories including (1) organisational factors affecting SDM and deprescribing, (2) moral injury of healthcare staff working in palliative care, and (3) characteristics of ATTs specific to deprescribing efforts. Ten papers were sought in each of these categories using RCQRS on the existing database. This process took 5 days and consisted of an additional 257 database records screened. As with the initial screening, these papers retrieved were entered into an appraisal journal in a co-productive process with the co-investigative team and then included in the rough analysis. A second set of stakeholder consultation meetings was undertaken to present progressed findings in month 10 of the 12-month review.

## 10 Step Five: Combine RCQRS with other literature identification approaches including hand searching and expert solicitation

RCQRS used with bibliographic database searches is best combined with other literature identification approaches. While the strategy allows teams to move quickly to data immersion and analysis, key papers within an older date range may be left out due to the quota being filled before reverse chronology screening reaches such papers. These papers may be important for understanding key drivers for the inception of programmes or innovations that have occurred in the past. Three additional strategies can support the inclusion of older, important papers. The first is systematic reference checking of relevant papers identified using RCQRS. Given that the papers retained in the database searching will be the most recent papers, reviewing the reference lists for each paper and selecting other papers through this process (backward citation searching) can ensure that other papers, including seminal papers in the area, are captured. Having a separate quota for evidence reviews can also support the retrieval of older, seminal papers. If such an approach is taken, a smaller initial quota size may be needed to ensure the feasibility of the dataset volume when any earlier published seminal papers are added. The second strategy is hand searching, in which the table of contents of academic journals or professional-body magazines is searched from the most recent edition in the series working backward until a quota is filled. This is particularly useful when applied to emerging journals not indexed in the bibliographic databases, but that are particularly relevant to the study area, or when quotas cannot be filled by database retrievals alone. The third strategy is retrieving papers through expert solicitation. This involves contacting the wider network of team members, project stakeholders, advisory groups, or consortium members to ask them to send the review team key papers of any date that match the review questions and the programme theories under scrutiny. Expert solicitation can be advantageous because experts can point to key seminal papers of the area of inquiry, which would likely be buried in database searches. These stakeholders can then send papers they have authored, as well as papers they recommend from other authors.

### 10.1 SERENITY reflection

RCQRS was combined with solicitation of papers from experts involved in the review. Thirty-five papers in total were recommended by members affiliated with the SERENITY project. Most of these papers involved biomedical content on the challenges associated with thrombotic complications in cancer patients and the need to optimise ATT prescribing. With a few exceptions, these papers had little content on SDM and strategies for deprescribing. However, they were supportive of the context-sensitive needs of the realist review, providing key understanding of the specific context of thrombosis and meanings patients have in relation to their thrombosis medications, through which the team was able to provide causal extrapolations in tandem with the general and diverse literature on deprescribing and SDM retrieved from the databases.

## 11 Discussion

RCQRS is an innovative screening strategy for realist synthesis that is needed at a time when realist methodology is rapidly expanding in many sectors, including the health services. Using RCQRS, teams can accomplish efficient and adaptable reviews in relatively short timeframes. Although ERS can be useful, the benefits reduce when the review scope narrows for reasons related primarily to evidence management. The result of such narrowing is that the pragmatic needs that the review is designed to address may be thwarted. Overlabouring the screening process prioritises relevance at the expense of richness. There may be surprising innovations that come from analysing a heterogeneous dataset, and this is missed when reviewers narrow the screening strategy for manageability-related reasons. The learnings gained by expert researchers when they read papers outside their narrowed area of specialisation can be particularly valuable. RCQRS allows for learnings at each stage of the review, which can shape the review scope iteratively. This is important for realist synthesis because of the difficulty of knowing the relevance and richness of papers^28^ from title and abstract screening alone.

Paradoxically, tangentially relevant papers that provide insights from other sectors and contexts may be more useful to stimulate innovative thinking and may enhance the realist analysis in ways that the highly relevant papers, closely matched to the inclusion criteria, do not. Highly relevant papers without adequate descriptions of programme architecture, contextual factors, and their causal insights can lead to ‘causally thin’ extrapolations. Alternatively, capturing a diverse set of papers in the screening process, including both exact matches and tangentially interesting papers, increases the creative extrapolation potential and the learnings that accompany this approach. Vetting insights from these papers with project stakeholders, including co-investigators, practitioners, and members of the public, early in the review can confirm the relevance of these insights and provide new directions for the subsequent stages of the process.

RCQRS has been developed in parallel with other strategies to reduce the screening time in systematic reviews^29^ and at a time of rapid shifts towards machine learning approaches in evidence syntheses.^30^ While automated approaches to ranking evidence on applications such as Covidence and Google Scholar have been in existence for some time, machine learning approaches are relatively new and are being used to manage increasingly large sets of records. Machine learning requires screening several hundred or even a few thousand citations to train the software to make appropriate automated inclusion decisions. Once this is complete, the AI tool can rapidly screen very large volumes of database records to retrieve papers that are specifically matched to the intervention area under investigation. Given the need for AI tools engage in bespoke ‘learning’ in advance of the screening process, RCQRS involving human cognition in every decision on records screening can offer a competitive advantage to machine learning in three ways. The first is that RCQRS can be conducted in short timeframes comparable to the setup and use of AI tools. Secondly, engaging reviewers during screening exposes their thinking to a broad range of published studies in and around the area of investigation. This exposure leads the reviewer to greater awareness of the scope of research being conducted in their fields, supporting their professional development and learning. Finally, unlike developments to automate the screening process, RCQRS requires relatively little preparatory work to set up and retains a diverse set of papers which, when analysed using holistic coding techniques, can yield fresh and surprising thinking about how to innovate and customise programmes addressing complexity and difficult problems. Having said this, using machine learning tools in conjunction with RCQRS could be beneficial or disadvantageous, and further research in this area is warranted.^31^ For example, training a tool to apply relevance ranking, would potentially override the chronological character of RCQRS, being organised by relevance rather than date. However, there is a concomitant risk of capturing mainly papers with close-to-exact relevance to the study scope without a focus on richness and insights from alternative fields, theoretical perspectives, or methods. Screening with machine learning on a relatively small batch of papers (e.g., until there were approximately 15 studies), may lead to missed opportunities for capturing heterogeneity in the literature, if the machine learning approach clustered similar studies before reviewers explore how different types of studies, interventions, and contexts may be beneficial for the realist analysis.

An important reflection stemming from the process of applying RCQRS in numerous studies is that, whereas relevance can largely be determined at title and abstract screening, richness can reasonably only be determined at full-text screening. RCQRS addresses this important challenge by permitting reviewers to begin analysing papers early in the review, offering insights for iterative modifications to the review scope and design. Seemingly minor statements in the introduction and discussion sections of papers may yield important insights into the overall analysis^32^ and causal explanatory insight is often retrieved in small sections of data in the literature (i.e., nuggets^33^) from a variety of data sources.^34^^,^ ^35^ There is no realist roadmap for finding these nuggets of insights. Realist reviewers need to heighten their sensitivity to causal statements and discover new ideas through the process.

In terms of possible disadvantages of using RCQRS, the approach does not invite a full screening of available database records, and as such may risk the possibility of missing suitable papers. It is for this reason that the approach should be conducted in tandem with other strategies for capturing relevant papers such as hand searching and expert solicitation. Even these additional approaches may have limitations, for example experts may only recommend papers that fit their assumptions or their body of work. Such recommendations may also bring papers to the analysis that have less relevance or richness than the review requires. Although these limitations may be offset by the benefits in terms of developing a rich and diverse set of papers, the approach should be explored carefully considering the needs of the review, human resources, and the timeframe afforded to its completion. Finally, RCQRS has been developed in consideration of realist methodology principles; however, it may have benefits for other types of evidence syntheses, including narrative and systematic reviews. Further exploration for expanding the approach to other areas is warranted.

## 12 Conclusion

RCQRS presents a novel approach to screening database records in a realist synthesis that optimises the time allocated for the review, thereby bringing a rich, diverse set of contemporaneous papers for understanding current practices. As realist synthesis relies on creative extrapolations as well as scientific rigour in the process, this technique holds the promise of optimising realist synthesis and delivering on its potential for health service research and beyond.

## Supporting information

10.1017/rsm.2025.10068.sm001 Jagosh et al. supplementary material Jagosh et al. supplementary material

## Figures and Tables

**Figure:**
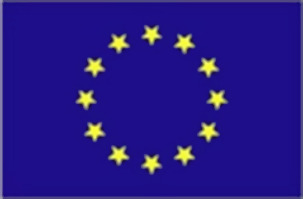


## Data Availability

Data availability is not applicable to this article as no new data were created or analysed in this study.

## Supplementary material

To view supplementary material for this article, please visit http://doi.org/10.1017/rsm.2025.10068.

## Long descriptions

### Table 1 Long description

The table is structured with a header row stating the example framing of a realist synthesis question: ‘What works, for whom, and in what circumstances for drug addiction recovery?’. Below this, the table is divided into two columns: ‘Narrowing to...’ and ‘Explanation’.

* Row 1: Narrowing to a subset of interventions. The explanation describes choosing one type of intervention, such as peer-led interventions, while excluding others like provider-led interventions.

* Row 2: Narrowing to a subset of contexts. The explanation describes focusing on a specific setting or demographic, such as drug addiction recovery for persons experiencing homelessness, while excluding other circumstances.

* Row 3: Narrowing to a subset of study design and literature source. The explanation describes retaining only specific designs like systematic reviews or R C T s. The example given is retaining only R C T s on drug addiction recovery for homeless populations while excluding case studies, qualitative research, and commentaries.

Navigate back to Table 1.

### Table 2 Long description

The table contains nine rows of definitions.

- Database Record. A collection of information about a published paper including title, abstract, authorship, date, journal of publication, and location.

- Database Record Library. The complete set of records retrieved from a database search after duplicates have been removed.

- Database Searching. The process of identifying key search terms and entering these into bibliographic databases which index records of published empirical and non-empirical papers.

- Exhaustive Record Screening. The process of reading and adjudicating on every record produced through the database search after duplicates are removed. E R S means all screening is complete before included records are used to retrieve and read the corresponding full-text paper.

- Expert Solicitation. Contacting experts including researchers, practitioners and service-users to recommend literature that they believe would be beneficial to be included in the review.

- Hand Searching. The process of selecting key academic journals, scanning the table of contents to identify potentially relevant papers for inclusion in the review.

- Reverse Chronology Quota Record Screening. The process of screening database records in reverse chronology from the most recent publication date until quotas of suitable papers have been filled.

- Screening. The process of reading database records and deliberating on the suitability of the records for including in the synthesis.

- Study Selection. The full set of strategies including screening and critical appraisal that allow reviewers to deliberate on and retain a group of papers for realist analysis.

Navigate back to Table 2.

### Table 3 Long description

The table is divided into two main sections: Advantages and Limitations.

Advantages section:

1. Improved pacing of review stages and estimations of time required to completion: Setting quotas for screening improves pacing and inspires reviewer confidence.

2. Increased time for data immersion and analysis: R C Q R S reduces screening time to 25 to 50 hours compared to 1000 hours for exhaustive approaches.

3. Reduced decision fatigue at screening stage: Reduces over-labouring when screening large volumes (greater than 15,000 records).

4. Improved representation of diversity in context and programme architecture through multiple quota categories: Ensures representation from under-researched areas.

5. Retention of papers that are roughly relevant to the review topic supports new discovery and creative insights: Tangential papers support abductive theorising and intellectual growth.

6. Studying Programmes in Most Recent Contexts, Circumstances and Settings: Captures recent publications and innovations for pragmatic utility.

Limitations section:

1. Potential to miss important papers after quotas are filled from the database record library: Only a portion of the library is screened; combining with other strategies is recommended.

2. Set of papers retained using R C Q R S is too diverse to analyse for specific review questions: May not be suitable for novice reviewers or very specific review focuses.

3. Combining R C Q R S with expert solicitation may introduce papers which are not relevant, or are too specific: Expert recommendations may not align with review goals, causing inefficiency.

Navigate back to Table 3.

### Figure 1 Long description

A central diagonal stack of paper icons represents a database search. The top-left end is labeled Oldest records and the bottom-right end is labeled Newest records retrieved in the database search. The process flows through five numbered steps.

* Step 1 at the bottom right. Determine the quota size for the group and subgroups of papers and fill the quotas. An icon shows a group of people with lightbulbs over their heads.

* Step 2 in the middle right. Retrieve and appraise full-text papers, and refill quotas when papers are eliminated upon full-text reading. An icon shows several document pages. A green arrow points from the newest records to this step.

* Step 3 at the top right. Produce a preliminary realist analysis and present early findings to stakeholders. An icon shows people presenting at whiteboards.

* Step 4 in the upper center. Return to R C Q R S screening to continue refilling quotas and for subsequent, specialized data retrieval. An icon shows three people working at computers. A blue arrow points from this step back to a blue-shaded section of the record stack.

* Step 5 at the middle left. Combine R C Q R S screening with other literature identification approaches including hand searching and expert solicitation. Icons show an open book, a group of people with a lightbulb, and a stack of books.

Navigate back to Figure 1.

### Table 4 Long description

The table consists of five rows, each detailing a specific step in the process.

Step 1. Determine the quota size or sizes for the numbers and screen records to the quota limits.

Step 2. Retrieve and appraise full-text papers, and refill quotas when papers are eliminated upon full-text reading.

Step 3. Produce a preliminary analysis and present early C M O configurations to stakeholders.

Step 4. Return to R C Q R S after preliminary analysis to continue refilling quotas and for specialised data retrieval.

Step 5. Combine R C Q R S with other literature identification sources including hand searching and expert solicitation.

Navigate back to Table 4.
