# Supplementary material for: Reverse chronology quota record screening for realist synthesis: Fostering causally rich extrapolations with a diverse and contemporaneous sample of literature
Source: Res Synth Methods. 2026 Jan 26;17(4):657–70. doi: 10.1017/rsm.2025.10068 (PMC13311346; doi:10.1017/rsm.2025.10068)
Supplement: Jagosh et al. supplementary material [file S1759287925100689sup001.docx]

**Appendix 1: Checklist for Using *Reverse Chronology Quota Record Screening* in Realist Synthesis**This checklist is meant to serve as a rough guide to determining quota and sub-quota sizes, along with considerations for quota categories. The checklist can be used during protocol development and to capture iterative modifications to the screening strategy as the review progresses. The checklist can be published as an appendix for improved transparency in reporting the process of retaining studies. There are no set rules for how many papers should be retained and reviewers should make final decisions based on their unique circumstances. The examples presented in the checklist serve to illustrate types of categories. Reviewers should develop categories in relation to the unique requirements of their review area.

| 1. **Quota Numbers:** | Number of months: | Estimate the total number of papers to be retained and provide justification: |
| --- | --- | --- |
| Enter Number of Months Allocated for the Review  Rough Guide:   - 3-month review (15-30 papers) - 6-month review (30-60 papers) - 12-month review (60-90 papers) - 18-month review (90-120 papers) | N= |  |
|  |  |  |
| **2. Quota Categories:** | Number of categories: | List quota categories for each section and retention numbers set for each quota category with rationale provided. (Quotas are not required for all sections): |
| 2a. Categories for key context aspects and variations:  Examples   - Healthcare setting (community, hospital) - Discipline (geriatrics, pediatrics) - Geography (rural, urban) - Population (general, minority) | N= |  |
|  |  |  |
| 2b. Categories for key programme aspects and variations:  Examples   - Modality (professional, peer-support) - Interface (live, technology-mediated) - Stakeholder (leaders, frontline workers) - Employment (direct, hub-and-spoke) | N= |  |
|  |  |  |
| 2c. Categories for key study designs:  Examples   - Qualitative research papers - Quantitative research papers - Reviews (systematic, narrative etc.) - Reports and Grey Literature | N= |  |
|  |  |  |
| **3. Screening Modifications or Additions:** | | List any modifications or additions to the quota numbers and categories determined after protocol registration or publication (for example, as a result of stakeholder input during data collection or data analysis). |
|  | |  |
